# Targeting NLRP3 Inflammasome and Metabolic Dysregulation by Electroacupuncture: A Novel Therapeutic Strategy for Methamphetamine Withdrawal-Induced Depression

**DOI:** 10.1080/17590914.2026.2624589

**Published:** 2026-02-13

**Authors:** Xiong Zhang, Xiao-Rui Zhao, Chun-Li You, Fang Zhang, Hong-Yi Zhu, Tian Gu, Jia Li

**Affiliations:** a College of Acumox and Tuina, Guizhou University of Traditional Chinese Medicine, Guiyang, Guizhou Province, China; b Teaching and Research Office of Traditional Chinese Medicine, Guizhou Nursing Vocational College, Guiyang, Guizhou Province, China; c Traditional Chinese Medicine Tumor Treatment Center, Chongqing University Cancer Hospital, Chongqing, China; d Medical Department, Kangxin Hospital of Guizhou Province, Guiyang, Guizhou Province, China; e Department of Nursing, Guizhou Nursing Vocational College, Guiyang, Guizhou Province, China

**Keywords:** Blood-brain barrier, depression, electroacupuncture, METH withdrawal, neuroinflammation, NLRP3

## Abstract

This study demonstrates that electroacupuncture (EA) produces robust antidepressant effects in a rat model of methamphetamine (METH) withdrawal. Behavioral tests showed that EA applied at GV20, PC6, and HT7 significantly reduced immobility in the forced swim test and enhanced exploratory activity in the open field test. Mechanistically, EA repaired blood–brain barrier (BBB) disruption, as shown by reduced hippocampal water content, decreased Evans Blue leakage, and restored expression of tight-junction proteins (Occludin, Claudin-5, ZO-1). EA also inhibited neuronal apoptosis, suppressed microglial activation, and lowered pro-inflammatory cytokines IL-6 and TNF-α. Multi-omics analyses revealed that EA reversed METH-induced alterations in 32 differentially expressed genes related to the NLRP3 inflammasome pathway (Nlrp3, Pycard, Il1b) and BBB function, while metabolomic profiling identified 13 key metabolites involved in glutamate metabolism, TCA cycle, and tryptophan pathways. Crucially, the therapeutic benefits of EA were abolished by intracerebroventricular administration of the NLRP3 activator nigericin, confirming the essential role of NLRP3 inflammasome inhibition in EA’s mechanism of action. In summary, EA represents a promising non-pharmacological approach for treating METH withdrawal-induced depression by coordinating BBB protection, suppression of neuroinflammation, and metabolic network regulation.

## Highlights

1. This study first reports that electroacupuncture alleviates METH-withdrawal depression by targeting the NLRP3 inflammasome pathway.

2. This study found that electroacupuncture significantly restores BBB integrity and reduces neuroinflammation in METH-withdrawn rats.

3. This study identified key transcriptomic changes (e.g., Nlrp3, Il1b) and metabolic alterations (glutamate, TCA, tryptophan) reversed by electroacupuncture.

4. This study demonstrated that the NLRP3 activator nigericin abolishes electroacupuncture’s therapeutic benefits, confirming pathway dependence.

5. This study provides a new theoretical basis for non-pharmacological intervention in substance withdrawal-induced depression.

## Graphical abstract

**Figure:**
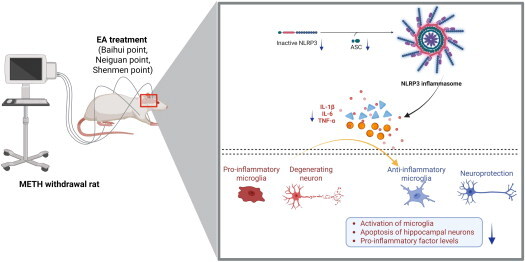
Molecular mechanism of EA-mediated regulation of the NLRP3 inflammasome pathway and BBB function in METH withdrawal-induced depression.Created in BioRender.

## Introduction

Methamphetamine (METH) is a highly addictive central nervous system (CNS) stimulant, and its abuse constitutes a serious global public health issue (Barr et al., 2006; Jones et al., 2020). Numerous studies have shown that long-term METH use induces not only neurotoxicity but also persistent emotional disorders such as depression and anxiety following withdrawal (Hosseini et al., 2023; Keshavarzi et al., 2019; Wang et al., 2025). Withdrawal depression induced by METH is often characterized by anhedonia, reduced spontaneous activity, and social withdrawal and is one of the main psychological factors contributing to relapse (Karabulut, 2024; Kudryavtseva et al., 2007; Xin et al., 2022). Current clinical interventions primarily rely on antidepressant medications; however, their efficacy is limited, side effects are common, and acceptance is low among individuals with substance dependence, resulting in unsatisfactory treatment outcomes (Zhang et al., 2024). Depression-like behavior after METH withdrawal involves not only neurotransmitter imbalances but also structural and functional brain abnormalities, immune activation, and other multisystem alterations, making its underlying mechanisms more complex than those of typical depression (Mo et al., 2024; Wang et al., 2025).

Growing evidence suggests that inflammation within the CNS plays a pivotal role in the development of addiction-associated depression, underscoring the therapeutic potential of targeting inflammatory mechanisms (He et al., 2023). Multiple studies have reported the link between depression, stress, and neuroinflammation. Damage-associated molecular patterns and CNS inflammation induced by chronic stress are often mediated through activated microglia. The NLRP3 inflammasome facilitates this process by releasing IL-1β, triggering a cascade of inflammatory responses (Chen et al., 2024). In addition to neuroinflammation, impairment of the blood–brain barrier (BBB) may also contribute to the emotional disturbances that emerge during withdrawal (Jiang et al., 2025). Therefore, investigating the mechanisms of neuroinflammation and BBB impairment in METH withdrawal depression is essential for understanding its pathological basis and identifying effective therapeutic strategies.

The BBB, composed of brain microvascular endothelial cells and their tight junctions, plays a critical role in preserving the brain’s internal homeostasis. Growing evidence shows that METH abuse severely compromises BBB integrity, leading to increased permeability and diminished levels of tight-junction proteins (including Occludin, Claudin-5, and ZO-1), thereby facilitating the entry of inflammatory cytokines into brain tissue (Mahajan et al., 2008; Martins et al., 2011). BBB disruption further activates microglia, promoting the release of pro-inflammatory mediators such as interleukin-6 (IL-6) and tumor necrosis factor-α (TNF-α), which intensify neuroinflammation and exacerbate neuronal injury (Jumnongprakhon et al., 2016). These pathological changes are closely associated with emotional disorders, suggesting that BBB dysfunction may constitute a critical pathological basis of withdrawal depression. The NLRP3 inflammasome has recently emerged as a key focus in neuroinflammation research (Bian et al., 2022). As a key cellular sensor of damage signals, activation of the NLRP3 inflammasome drives the release of the pro-inflammatory cytokines IL-1β and IL-18. METH-induced oxidative stress and cellular injury can trigger this pathway, initiating an inflammatory cascade that contributes to neuronal damage and behavioral disturbances (Hui et al., 2024). In depression research, the NLRP3 inflammasome has also been shown to be strongly associated with mood disorders (Jiang et al., 2022; Kouba et al., 2022). However, whether the NLRP3 inflammasome is directly involved in BBB disruption and emotional disturbances following METH withdrawal remains to be fully elucidated. Clarifying the specific role of this pathway may offer new therapeutic strategies for addiction-related depression. In addition to these pathological alterations, increasing evidence indicates that the hippocampus is one of the brain regions most vulnerable to METH-induced injury. As a key structure involved in emotional regulation, stress responsivity, and depression-related neuroplasticity, the hippocampus is tightly linked to the affective disturbances observed during METH withdrawal (Bartsch and Wulff, 2015; McEwen, 2017; Schmaal et al., 2016). Previous studies have shown that METH exposure leads to significant hippocampal abnormalities, including neuronal apoptosis, microglial activation, and disruption of tight junction proteins essential for maintaining BBB integrity (Gonçalves et al., 2017; Hajheidari et al., 2017; Ohene‐Nyako et al., 2021; Shahidi et al., 2019). Consistent with these observations, preliminary findings from our laboratory also revealed marked structural and inflammatory changes within the hippocampus following METH withdrawal, highlighting this region as a critical target of METH-related neurotoxicity. Given its functional significance and heightened vulnerability, the hippocampus was therefore selected as the primary region for molecular, histological, and multi-omics analyses in the present study.

Electroacupuncture (EA), a treatment combining acupuncture with electrical stimulation, has been widely applied in traditional Chinese medicine (TCM) for neuropsychiatric interventions. Previous research has demonstrated that EA can modulate the release of neurotransmitters such as dopamine and serotonin, mitigate depression- and anxiety-like behaviors, and exert both anti-inflammatory and neuroprotective effects (Han et al., 2018; Zhou et al., 2022). In conventional animal models of depression, EA has shown significant improvements in both behavioral parameters and neuroinflammatory markers (Armour et al., 2019; Lin et al., 2020). Notably, stimulation of specific acupoints, such as Baihui (GV20), Neiguan (PC6), and Shenmen (HT7), has yielded clear regulatory effects on central emotional activity. Compared with pharmacological treatment, EA offers greater clinical potential due to its minimal side effects, better compliance, and suitability for individuals with substance dependence. However, direct evidence is still lacking regarding whether EA can alleviate depression-like symptoms following METH withdrawal and whether its effects involve modulation of the NLRP3 inflammasome or restoration of BBB function. The application of multi-omics techniques, including RNA sequencing (RNA-seq) and metabolomics, has provided powerful tools to uncover the underlying mechanisms of EA (Liu et al., 2022). By systematically evaluating gene expression and metabolic alterations, it is possible to identify key regulatory pathways involved in the therapeutic process comprehensively. Integrating multi-omics with TCM-based interventions offers a modern explanation of traditional practices and provides a theoretical foundation for precision medicine strategies.

Building on this background, the present study sought to determine whether EA can ameliorate depression-like behaviors in rats undergoing METH withdrawal and to clarify whether its therapeutic effects are linked to modulation of the NLRP3 inflammasome and restoration of BBB function. By integrating assessments of neuroinflammation, barrier integrity, and emotional behavior, this research aimed to construct a rigorous mechanistic framework for understanding the pathophysiology of METH withdrawal–induced depression. The findings are expected to reveal potential therapeutic targets for METH-related mood disorders and deepen our understanding of addiction-associated neuropathology. Moreover, this study provides theoretical and experimental support for applying EA, an important modality in TCM, to addiction-related psychiatric conditions. Clinically, if EA proves effective in alleviating emotional disturbances and repairing CNS damage, it may offer a safe, practical, and low-dependence treatment option for individuals affected by substance abuse, carrying meaningful implications for public health.

## Materials and Methods

### Establishment of the METH Withdrawal Rat Model

Healthy adult male Sprague–Dawley rats (8–10 weeks, 180–220 g) were obtained from Beijing Vital River Laboratory Animal Technology Co., Ltd. (Beijing, China). Animals were housed under standard laboratory conditions (22 ± 1 °C; 50 ± 5% humidity; 12-h light/dark cycle) with free access to food and water. All procedures complied with the National Guidelines for the Use of Laboratory Animals and were approved by the Institutional Animal Care and Use Committee (Approval No.: gzhlllscb2024-0201).

After a 3-day acclimation period, baseline behavioral assessments were conducted using the forced swim test (FST) and open field test (OFT). Rats with comparable baseline scores were selected for model induction. METH was dissolved in 0.9% saline (1 mg/ml) and delivered via intraperitoneal injection at 2 mg/kg, while control rats received saline (1 ml/kg). The dose of 2 mg/kg METH was selected based on previous studies showing that this dosage effectively induces robust conditioned place preference (CPP) and subsequent withdrawal symptoms without causing severe stereotypic behaviors or toxicity that might interfere with behavioral testing (Havlickova et al., 2018; Shabani et al., 2011). Although species differences in metabolism exist, this dose is widely accepted to mimic the psychostimulant and rewarding effects relevant to human METH abuse. The CPP test was used to establish the METH addiction model through four phases: habituation, pre-conditioning, conditioning, and post-conditioning.

CPP Test: The apparatus consisted of a three-chamber shuttle box, including two conditioning chambers of equal size (70 cm × 30 cm × 34 cm; length × width × height) and a central chamber (13 cm × 22 cm × 27 cm). When the partition was inserted, rats were confined to a specific chamber; when removed, they were allowed to move freely among all three chambers. The time spent in each chamber was recorded to assess the rats’ place preference behavior.

① Habituation Phase: Rats were placed in the shuttle box with the partitions removed and allowed to freely explore all compartments for 15 minutes daily over three consecutive days. This was done to reduce novelty-induced stress and the influence of environmental unfamiliarity.

② Pre-conditioning Phase: Before conditioning began, rats were placed in the shuttle box with the partitions removed and allowed to move freely for 15 minutes. The time spent in each conditioning chamber was recorded. If a rat showed a natural preference—spending more than 150 seconds longer in one chamber compared to the other—it was excluded from the study.

③ Conditioning Phase: One conditioning chamber was designated as the drug-paired side, with light cues provided; the other was designated as the non-drug-paired side, without light cues. Starting the day after the pre-conditioning test, rats underwent alternate-day conditioning sessions. On Day 1, rats received an intraperitoneal injection of METH at 5 mg/kg and were confined to the drug-paired chamber for 15 minutes. On Day 2, they received an injection of saline (1 ml/kg) and were confined to the non-drug-paired chamber for 15 minutes. This alternating training protocol was repeated for 28 days.

④ Post-conditioning Phase: Twenty-four hours after the final conditioning session, rats were placed in the central compartment of the shuttle box with the partitions removed and allowed to move freely for 15 minutes. The time spent in each of the two conditioning chambers was recorded.

After the METH addiction model was successfully established using the CPP protocol, rats underwent FST and OFT assessments, followed by a 10-day period of natural withdrawal. Behavioral testing was then repeated. Depression-like behavior was determined by comparing pre- and post-withdrawal data. Rats showing a significant increase in FST immobility time, together with reduced central zone activity, decreased rearing, and lower total locomotor scores in the OFT, were classified as having withdrawal-induced depression. These criteria confirmed the successful establishment of the METH withdrawal depression model.

### EA Treatment

After confirming withdrawal-induced depression, rats were randomly assigned to five groups (n = 8 per group): the control group, the METH withdrawal group (Model), the METH withdrawal + sham EA group (sham-EA), the METH withdrawal + EA group (EA), and the METH withdrawal + fluoxetine group (FLX).

In the EA group, EA was administered at Baihui (GV20), bilateral Neiguan (PC6), and Shenmen (HT7) acupoints based on anatomical references for humans and animals from the *Experimental Acupuncture Science* manual. After routine disinfection of the acupoints, 0.30 × 13 mm acupuncture needles were inserted, and once the “Deqi” sensation was obtained, the needles were connected to a 6805-II EA instrument. Continuous waves were applied with 5 ms pulse widths at a frequency of 100 Hz, adjusted to induce slight visible twitching of the local skin and muscle at the acupoint sites. The stimulation intensity was modulated to produce mild muscular tremors (approximately 1.5–2 mV) (Ho et al., 2017). Treatments were administered once daily for 15 minutes per session, continuously for 28 days.

In the sham-EA group, the same acupoints (Baihui (GV20), bilateral Neiguan (PC6), and Shenmen (HT7)) were selected based on the same anatomical references. After standard disinfection, acupuncture needles (0.30 × 13 mm) were shallowly inserted subcutaneously, and although connected to the EA instrument, no electrical stimulation was applied. This procedure was conducted once daily for 15 minutes over 28 days.

In the FLX group, rats were administered FLX hydrochloride suspension orally via gavage. FLX was dissolved in distilled water to a final concentration of 10 mg/kg and administered at a dose of 2.8 ml/kg based on body weight once daily for 28 consecutive days.

### Intracerebroventricular Injection

Intracerebroventricular injections were performed in mice using a stereotaxic apparatus (RWD Life Science, China). One day before the experiment, mice were anesthetized with 2% sodium pentobarbital (40 mg/kg, i.p.; Sigma-Aldrich, USA), positioned in the stereotaxic frame, and the scalp was disinfected. A small cranial opening was drilled, and a microsyringe was inserted according to the following coordinates relative to bregma: anteroposterior −0.22 mm, mediolateral 1.0 mm, dorsoventral −2.5 mm. Two hours before EA treatment, nigericin (20 μM; Sigma-Aldrich, USA) or normal saline (Beyotime, China) was infused using a microinjection pump (Harvard Apparatus, USA) at 0.5 μL/min for a total volume of 5 μL. The needle was retained for 5 minutes to prevent reflux. Following surgery, mice were placed in a 37 °C thermostatic chamber (Harvard Apparatus, USA) to recover and given free access to food and water. Animals were randomly assigned to three groups (n = 8 per group): METH withdrawal + EA (EA group), METH withdrawal + EA + vehicle (EA + vehicle group), and METH withdrawal + EA + nigericin (EA + nigericin group).

### OFT

The open field apparatus consisted of a square box (80 × 80 × 40 cm) under low-light conditions. Prior to testing, rats were placed in the testing room for a 6-minute acclimation period. During the test, horizontal and vertical locomotor activities were recorded in a field divided into 25 equal squares. A horizontal movement was counted when a rat crossed into a new square (1 point), and a vertical movement was recorded when both forepaws were lifted off the ground simultaneously (1 point). The duration spent in the central area was also measured. The arena was cleaned with 75% ethanol between tests.

### FST

Rats were individually placed in a cylindrical container filled with clean water (60 cm diameter × 60 cm height), with a water depth of 30 cm and temperature maintained at 25 ± 1 °C. Each rat was subjected to a 6-minute swimming session, and the duration of immobility during the final 4 minutes was recorded using a video tracking system. Immobility was defined as the absence of active escape behaviors, with the animal floating passively without struggling or marked body movements.

### Dry-Wet Method for Determination of Hippocampal Water Content

At the conclusion of the experiment, hippocampal tissue was rapidly dissected and weighed to determine its wet weight (W_wet_). Samples were then dried at 105 °C for 48 hours (Thermo Fisher Scientific, USA) to obtain the dry weight (W_dry_). Hippocampal water content was calculated using the formula: Water content (%) = (W _wet_ - W _dry_)/W _wet_ × 100%.

### Evans Blue Extravasation Assay

The left femoral vein was disinfected with 75% ethanol, and a 2% Evans Blue (EB) solution was administered at 4 ml/kg, followed by ligation of the vein. After 2 hours, rats were perfused transcardially with normal saline until the outflow became clear. Brains were collected by decapitation, and the tissue was weighed. Formamide was added at 2 ml per gram of tissue, and samples were incubated at 37 °C for 72 hours in the dark. The supernatant was then collected, and absorbance was recorded at 620 nm. EB concentration was calculated from a standard curve and expressed as μg/g of brain tissue.

### Immunofluorescence and TUNEL Staining

Brain sections were blocked for 1 hour at room temperature with 5% bovine serum albumin and 0.1% Triton X-100, then incubated overnight at 4 °C with primary antibodies against Occludin (ab216327, 1:200, Abcam), Claudin-5 (AF5216, 1:500, Affinity Biosciences), ZO-1 (61-7300, 1:200, Invitrogen), and Iba-1 (ab178846, 1 µg/mL, Abcam). The next day, sections were washed in (10010023, Invitrogen, USA) and incubated for 2 hours at room temperature in the dark with Alexa Fluor® 647-conjugated goat anti-rabbit IgG H&L secondary antibody (ab150079, Abcam, UK). After washing, nuclei were counterstained with DAPI (D9542, Sigma-Aldrich, USA).

For NeuN/TUNEL double labeling, sections were incubated overnight at 4 °C with an anti-NeuN antibody (ab177487, 1:100, Abcam), followed by detection of apoptotic cells using a TUNEL kit (Roche, USA). TUNEL-positive neurons were counted in the cortex by an independent observer. Images were acquired using a fluorescence microscope (Leica Microsystems, DMi8, Germany). ImageJ software was used to analyze microglial morphology, including the number of microglia, average number of endpoints per cell, and average total branch length per cell, to assess microglial activation status and morphological changes.

### Western Blot (WB)

Hippocampal tissue was lysed in RIPA buffer (RP20036, Proteintech, China), and protein extracts were collected by centrifugation at 13,000 × g for 10 minutes. Total protein concentration was determined using a BCA assay (23225, Thermo Fisher Scientific, USA). Equal amounts of protein (20 µg per sample) were separated on 10% SDS-PAGE gels (M00654, Gene Script, China) and transferred to PVDF membranes (3010040001, Millipore, USA). Membranes were blocked with 5% non-fat milk (P0216, Beyotime, China) for 1 hour at room temperature and incubated overnight at 4 °C with primary antibodies against Occludin (ab216327, 1:1000, Abcam, UK), Claudin-5 (AF5216, 1:500, Affinity Biosciences), ZO-1 (61-7300, 1 µg/mL, Invitrogen, USA), NLRP3 (30109-1-AP, 1:1000, Proteintech, China), ASC (DF6304, 1:500, Affinity Biosciences), IL-1β (ab315084, 1:1000, Abcam, UK), and β-Actin (20536-1-AP, 1:5000, Proteintech, China). The next day, membranes were washed and incubated for 1 hour with an HRP-conjugated goat anti-rabbit IgG secondary antibody (SA00001-2, 0.2 mg/mL, Proteintech, China). Protein bands were visualized using enhanced chemiluminescence (ECL, 32209, Thermo Fisher Scientific, USA) and quantified with the ChemiDoc XRS+ imaging system (Bio-Rad, USA).

### Transmission Electron Microscopy (TEM)

TEM was performed using a Hitachi HT7800 microscope (Japan) to observe the ultrastructure of hippocampal neurons. The hippocampal tissue was first dissected under a stereomicroscope and fixed in a 2% paraformaldehyde and 2.5% glutaraldehyde solution (BL910A, Biosharp, China) for 24 hours. Ultrathin sections were prepared using an ultramicrotome (Leica UC7, Germany) and examined under the TEM.

### ELISA

Blood samples were collected, centrifuged to obtain serum, and stored at −80 °C. Hippocampal tissue was dissected, homogenized in RIPA buffer, and centrifuged to collect the supernatant for protein quantification. TNF-α (CER1393, CRK Pharma, Wuhan) and IL-6 (CER0042, CRK Pharma, Wuhan) levels were measured using commercial ELISA kits.

### Bulk RNA-Seq and Data Processing

Rats were euthanized by cervical dislocation, and hippocampal tissue from the Control, Model, and EA groups (n = 3 per group) was collected. Total RNA was extracted using TRIzol (15596026, Thermo Fisher Scientific). RNA concentration and purity were assessed by NanoDrop ND-1000 spectrophotometer (OD260/280), and RNA quantity was also measured using the Qubit RNA Assay Kit (N608303-0100, Sangon Biotech). Samples were included for library preparation only if the RNA integrity number (RIN) was ≥ 7.0 and the 28S:18S rRNA ratio was ≥ 1.5.

For each sample, 5 μg of total RNA was used. Ribosomal RNA was removed with the Ribo-Zero™ Magnetic Kit (MRZH116, Epicentre Technologies, USA). Libraries were generated using the NEBNext Ultra RNA Library Prep Kit (E7770, New England Biolabs). RNA fragmentation was performed in NEBNext First Strand Synthesis Reaction Buffer (5×) to produce ∼300 bp fragments, followed by first- and second-strand cDNA synthesis (with dUTP incorporation into the second strand). cDNA fragments were then end-repaired, polyadenylated, and ligated to Illumina adaptors. USER enzyme (M5505S, New England Biolabs) was used to selectively degrade the second strand, yielding a strand-specific library. Libraries were amplified, purified, and evaluated using an Agilent 2100 Bioanalyzer, with final quantification performed using the KAPA Library Quantification Kit (TAQDKB).

Paired-end sequencing was conducted on the Illumina NextSeq CN500 platform. Raw reads were processed with Trimmomatic to remove adaptors, filter low-quality reads, trim poor-quality bases, and generate summary statistics including raw and effective read counts, Q30 values, GC content, and retention rates. High-quality reads were aligned to the Rattus norvegicus reference genome (NCBI) using Hisat2. Differential expression analysis was performed in R using the edgeR package. Genes with |log_2_ fold change| > 1 and *p* < 0.05 were classified as differentially expressed.

### Gene Ontology (GO) and Kyoto Encyclopedia of Genes and Genomes (KEGG) Pathway Enrichment Analysis

Functional enrichment of intersecting genes was conducted using the ClusterProfiler package (v4.2.2). GO analysis covered biological processes (BP), cellular components (CC), and molecular functions (MF). *p*-values were corrected using the Benjamini–Hochberg method, with significance defined as *p*.adjust < 0.05. Enrichment results were visualized as bubble plots generated with ggplot2 (v3.3.6).

### Metabolomics Sample Preparation and Liquid Chromatography-Mass Spectrometry (LC-MS) Analysis

Hippocampal tissue (50 mg) was homogenized in 800 µL of pre-cooled methanol/water (4:1, v/v; Sigma-Aldrich) using a TissueLyser II (Qiagen, Germany) at 30 Hz for 2 minutes. Homogenates were incubated at −20 °C for 1 hour and centrifuged at 14,000 × g for 15 minutes at 4 °C. The supernatant was collected for LC-MS analysis. Metabolite separation was performed using ultra-performance liquid chromatography (UPLC, ACQUITY UPLC H-Class, Waters, USA). The mobile phase consisted of 0.1% formic acid in water (Phase A) and 0.1% formic acid in acetonitrile (Phase B, Sigma-Aldrich, USA). Samples were injected (3 µL) onto an ACQUITY UPLC HSS T3 column (2.1 × 100 mm, 1.8 µm; Waters) at a flow rate of 0.4 mL/min. Mass spectrometry was performed on an Orbitrap Exploris 240 system (Thermo Fisher Scientific, USA) in both positive ion (ESI^+^) and negative ion (ESI^-^) modes, with a mass scan range of m/z 100-1000 and a resolution of 120,000.

The MS parameters were: ionization voltage 5500 V, capillary temperature 550 °C, sheath gas 50 psi, and auxiliary gas 60 psi. Data preprocessing and overfitting prevention were conducted using orthogonal partial least squares discriminant analysis (OPLS-DA) and permutation testing. Metabolites with a VIP > 1 and p < 0.05 were considered differential metabolites (DMs). Metabolic pathway enrichment was performed using MetaboAnalyst 5.0.

### Statistical Analysis

All data were analyzed using Prism statistical software (version 10.1.2). Continuous variables are expressed as mean ± standard deviation (SD). Independent-sample t-tests were used for two-group comparisons, and one-way ANOVA was applied for multiple-group comparisons. Categorical data were presented as rates or percentages and analyzed using the chi-square (χ^2^) test. A *p*-value < 0.05 was regarded as statistically significant.

## Results

### Successful Establishment of a Depression Model in Rats Following METH Withdrawal

**Figure 1.**
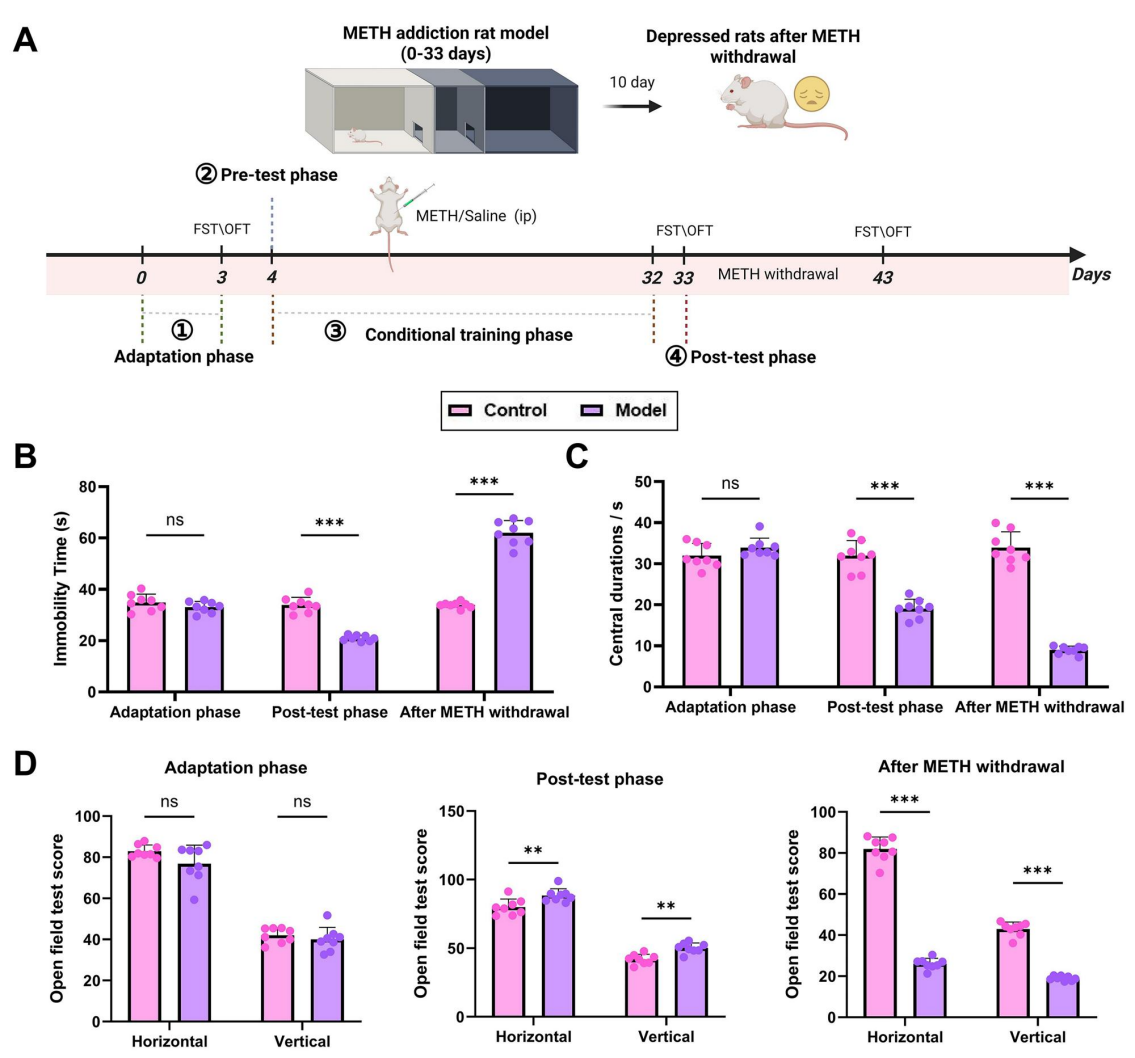
Establishment and validation of the depression model in rats following METH withdrawal. (A) Flowchart illustrating the construction of the depression model after METH withdrawal (Created in BioRender); (B) Immobility time during the FST in the adaptation phase, post-conditioning phase, and after METH withdrawal; (C-D) OFT assessing central zone activity duration (C), rearing frequency, and total locomotor score (D) in the adaptation phase, post-conditioning phase, and after METH withdrawal. N = 8; **p < 0.01; ***p < 0.001 compared between groups.

We established a METH addiction model in rats using the CPP test, which included four stages: habituation, pre-conditioning, conditioning, and post-conditioning. After model establishment, METH administration was discontinued, and rats underwent a 10-day period of forced withdrawal to induce depression-like behavior, thereby establishing a post-withdrawal depression model (see flowchart in Figure 1A). Depression-like behavior during the habituation, post-conditioning, and withdrawal phases was evaluated using the FST and OFT. In the FST, no significant difference in immobility time was observed between the Control and Model groups during habituation. However, during the post-conditioning phase, the Model group exhibited a significant reduction in immobility time, likely due to METH-induced central excitability and hyperactivity. After METH withdrawal, immobility time in the Model group was significantly prolonged, suggesting a depression-like phenotype, although the potential contribution of physical weakness cannot be fully excluded (Figure 1B).

In the OFT, no significant differences were observed between the Control and Model groups in central zone activity time, rearing frequency, or total locomotor scores during the habituation phase. During the post-conditioning phase, the Model group showed reduced time spent in the central zone but increased rearing and total locomotor scores, which may reflect METH-induced hyperactivity or anxiety-driven avoidance of the central area. After withdrawal, the Model group demonstrated a further pronounced reduction in central zone activity, accompanied by significant decreases in rearing behavior and total locomotor scores, indicating a robust depressive phenotype (Figure 1C-D).

In summary, we successfully established a rat model of depression following METH withdrawal.

### EA Effectively Alleviates Depression-Like Behavior and Restores BBB Function in Rats following METH Withdrawal

**Figure 2.**
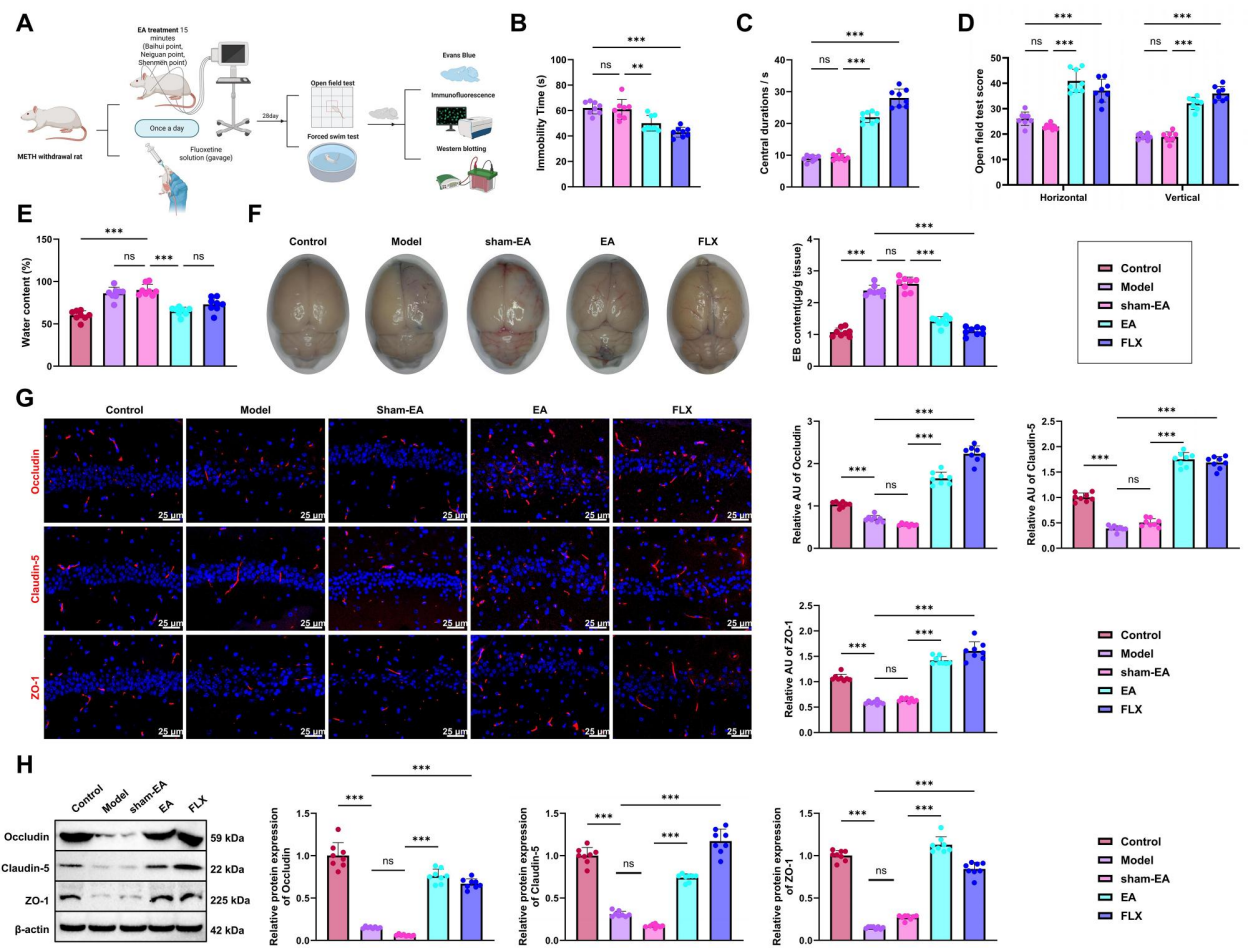
Effects of EA on depression-like behavior and BBB function in rats after METH withdrawal. (A) Flowchart of the EA, targeting Baihui (GV20), bilateral Neiguan (PC6), and Shenmen (HT7) acupoints (Created in BioRender); (B) Immobility time during the FST; (C-D) OFT results showing central zone activity duration (C), rearing frequency, and total locomotor score (D); (E) Hippocampal water content measured by the dry-wet method; (F) Evans Blue extravasation assay evaluating BBB permeability; (G) Immunofluorescence detection of BBB structural proteins Occludin, Claudin-5, and ZO-1 in the hippocampus; (H) Western blot analysis of Occludin, Claudin-5, and ZO-1 protein levels in hippocampal tissue. N = 8; *p < 0.05; **p < 0.01; ***p < 0.001 compared between groups.

To evaluate the therapeutic effects of EA on METH withdrawal–induced depression, we established a rat model of post-withdrawal depression. EA treatment was applied at Baihui (GV20), bilateral Neiguan (PC6), and Shenmen (HT7) acupoints, selected based on anatomical references for humans and animals (Figure 2A). After 28 days of intervention, behavioral assessments were conducted. In the FST, both the EA and FLX groups showed markedly reduced immobility times compared with the Model group, whereas the sham-EA group exhibited no significant change (Figure 2B). In the OFT, rats receiving EA or FLX exhibited significantly increased central zone activity, rearing behavior, and total locomotor scores relative to the Model group, while the sham-EA group showed no notable improvement (Figure 2C-D).

Hippocampal water content was then measured using the dry–wet method. The Model group displayed a pronounced increase relative to the Control group, whereas this rise was significantly attenuated in both the EA and FLX groups. No meaningful alteration was detected in the sham-EA group (Figure 2E). The Evans Blue extravasation assay revealed that Evans Blue permeability was markedly elevated in the brain tissue of the Model group relative to the Control group. In contrast, Evans Blue levels were significantly reduced in the EA and FLX groups compared to the Model group, with no notable difference observed in the sham-EA group (Figure 2F). Immunofluorescence and WB analyses revealed that the tight junction proteins Occludin, Claudin-5, and ZO-1 were significantly downregulated in the hippocampus of the Model group relative to the Control group. EA and FLX treatment substantially restored the expression of these tight-junction proteins, whereas the sham-EA group exhibited no notable changes (Figure 2G-H).

Taken together, EA intervention mitigated these behavioral deficits associated with the depression-like state and improved BBB function in rats following METH withdrawal.

### EA Effectively Inhibits Neuronal Apoptosis, Microglial Activation, and Inflammatory Cytokine Levels in Rats following METH Withdrawal

**Figure 3.**
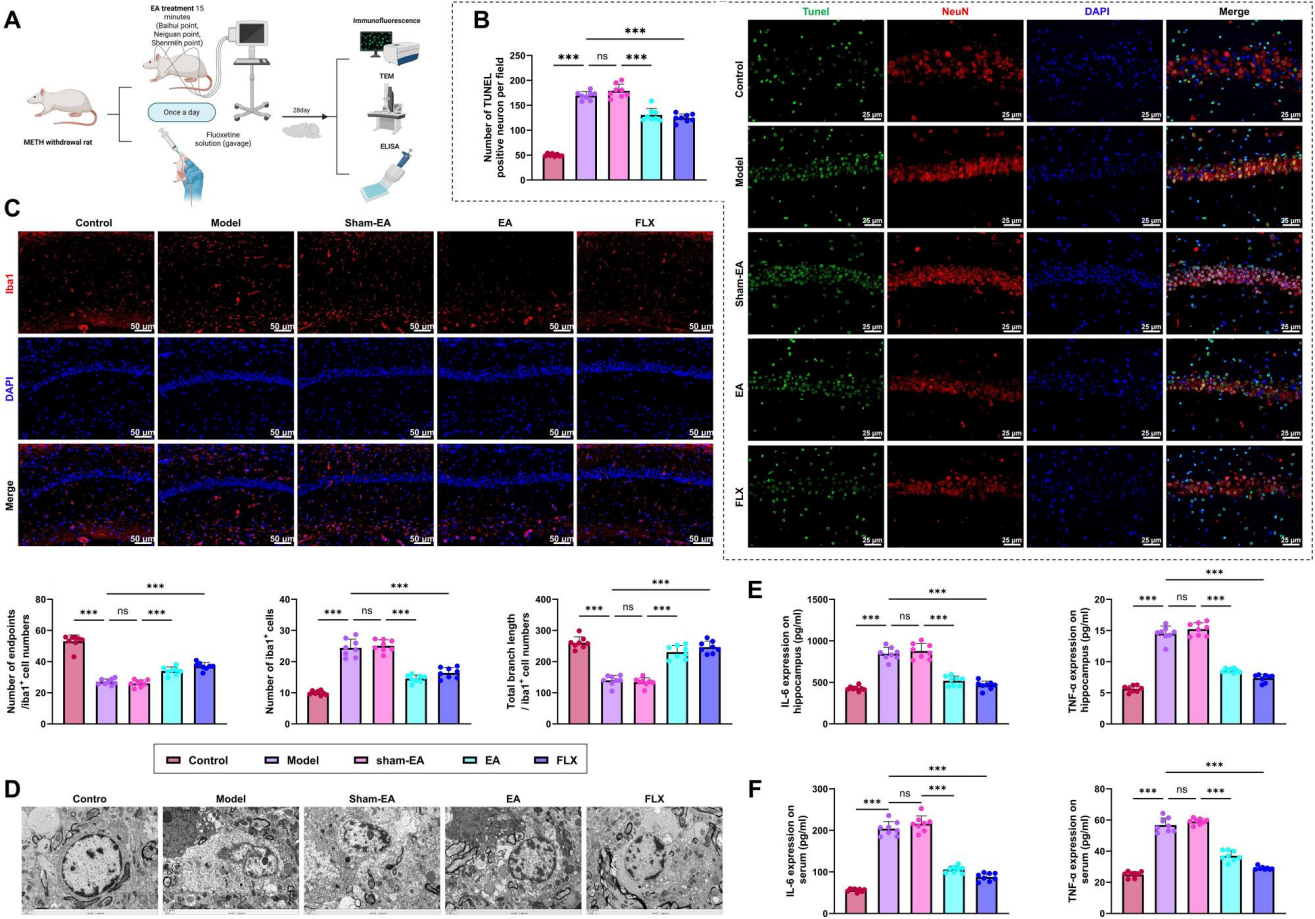
Effects of EA on neuronal apoptosis, microglial activation, and inflammatory cytokine levels in rats with depression following METH withdrawal. (A) Schematic diagram of the neuroprotective effects of EA in METH withdrawal rats (Created in BioRender); (B) TUNEL staining combined with NeuN immunofluorescence to assess hippocampal neuronal apoptosis; scale bar: 25 μm; (C) Iba-1 immunofluorescence staining to evaluate the number and morphological features of microglia in the hippocampus, including number of endpoints and total branch length; scale bar: 25 μm; (D) TEM for ultrastructural observation of hippocampal neurons; scale bar: 2 μm; (E-F) ELISA results for IL-6 and TNF-α levels in hippocampal tissue and peripheral serum. N = 8; ***p < 0.001 between groups.

We further investigated the effects of EA on neuronal apoptosis, microglial activation, and inflammatory cytokine levels in rats following METH withdrawal (Figure 3A). Neuronal apoptosis was evaluated using TUNEL staining combined with NeuN co-labeling. The Model group showed a marked increase in TUNEL-positive hippocampal neurons compared with the Control group, whereas both EA and FLX treatment significantly lowered the number of apoptotic cells. The sham-EA group exhibited no meaningful change. However, it is worth noting that the number of apoptotic neurons in both the EA and FLX groups remained significantly higher than that in the Control group (*p* < 0.001), indicating that while the intervention significantly mitigated neuronal damage, it did not fully restore it to baseline levels (Figure 3B). Immunofluorescence analysis revealed a substantial increase in Iba-1-positive microglia in the Model group, which was significantly reversed by EA and FLX treatment but unchanged in the sham-EA group. Morphological assessments further indicated that the Model group displayed a pronounced decrease in the average number of endpoints and total branch length of microglial processes compared with Controls. These alterations were significantly restored in the EA and FLX groups, with no improvement in the sham-EA group (Figure 3C). Additionally, TEM revealed ultrastructural damage in hippocampal neurons of the Model group, including nuclear pyknosis, mitochondrial swelling, and disrupted cristae, compared to the Control group. These pathological changes were notably alleviated in the EA and FLX groups, whereas the sham-EA group exhibited no significant improvement (Figure 3D).

ELISA results revealed that IL-6 and TNF-α concentrations were markedly elevated in both hippocampal tissue and serum in the Model group relative to the Control group. These increases were substantially reduced following EA or FLX treatment, while the sham-EA group showed no significant alterations (Figure 3E-F).

In conclusion, EA effectively inhibited neuronal apoptosis, attenuated microglial activation, preserved neuronal ultrastructure, and reduced IL-6 and TNF-α levels in rats following METH withdrawal. These effects may be attributed to its neuroprotective and anti-inflammatory properties.

### Multi-Omics Analysis Revealed That EA Effectively Restored BBB Function and Ameliorated Neuroinflammation-Associated Metabolic Dysregulation, Contributing to Neuroprotection

**Figure 4.**
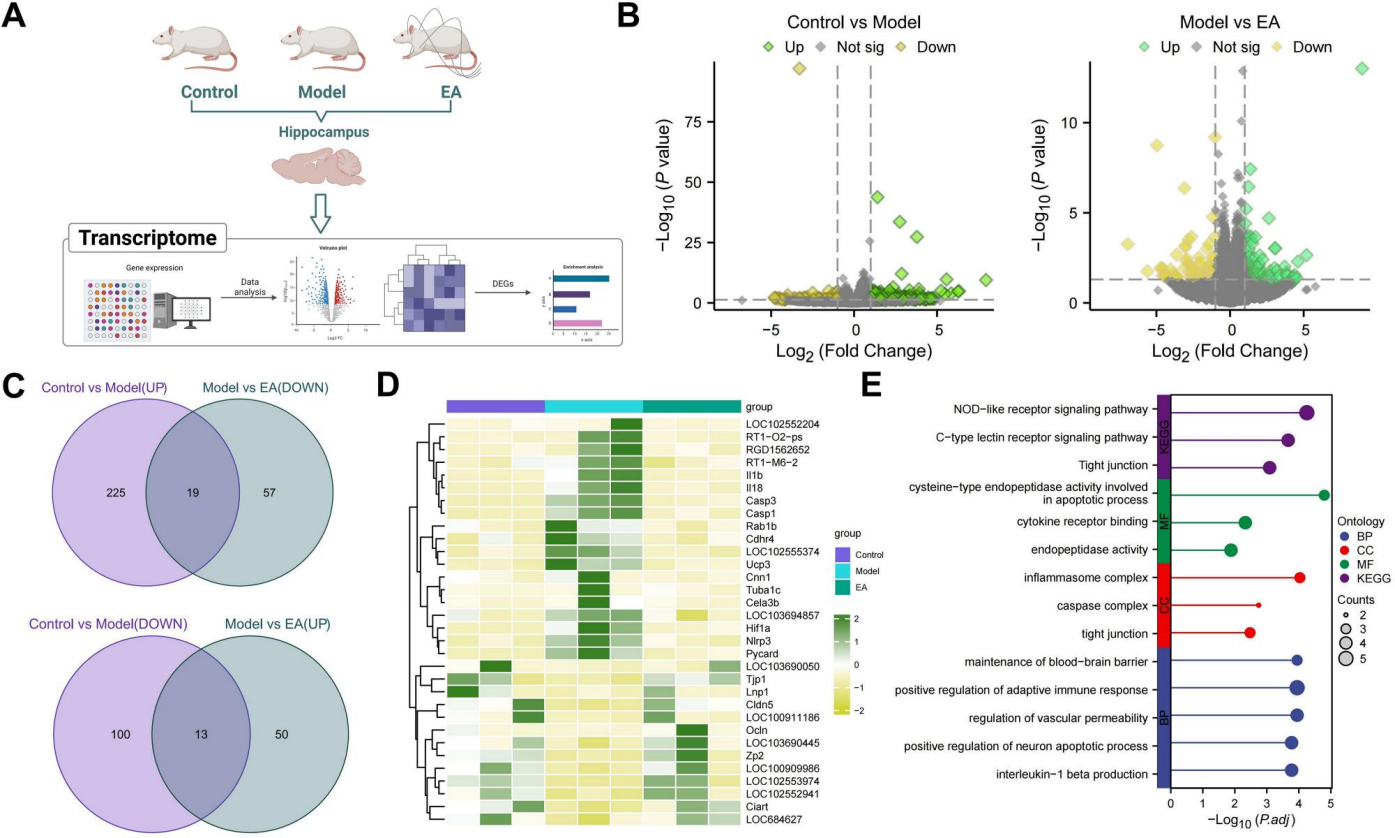
RNA-seq analysis of the potential mechanisms underlying the antidepressant effects of EA in METH withdrawal rats. (A) Schematic diagram of the RNA-seq workflow; N = 3 (Created in BioRender); (B) Differential expression analysis between the Control and Model groups, and between the Model and EA groups; (C) Identification of intersecting genes with reversed expression patterns following EA; (D) Heatmap showing expression profiles of 32 intersecting genes across all groups; (E) GO and KEGG enrichment analyses of intersecting genes.

To explore the specific mechanisms by which EA alleviates depression in rats following METH withdrawal, total RNA was extracted from hippocampal tissues of the Control, Model, and EA groups for RNA-seq analysis (Figure 4A). Differential expression analysis identified 3,704 differentially expressed genes (DEGs) between the Control and Model groups, including 2,055 upregulated and 1,649 downregulated genes in the Model group. Comparison between the Model and EA groups identified 189 DEGs, of which 113 were upregulated and 76 were downregulated in the Model group (Figure 4B). We then identified intersecting genes by overlapping the genes upregulated in the Control vs. Model comparison with those downregulated in the Model vs. EA comparison, and vice versa. This yielded 19 and 13 intersecting genes, respectively (Figure 4C). A heatmap illustrating the expression patterns of the 32 intersecting genes across the three groups (Figure 4D) revealed pronounced alterations in BBB-associated structural genes—Occludin (*Ocln*), Claudin-5 (*Cldn5*), and ZO-1 (*Tjp1*)—as well as inflammation-related genes in the NLRP3 inflammasome pathway—NLRP3 (*Nlrp3*), ASC (*Pycard*), and IL-1β (*Il1b*).

We subsequently conducted GO and KEGG enrichment analyses on these intersecting genes (Figure 4E). In the BP category, GO terms were predominantly related to BBB integrity, activation of adaptive immune processes, regulation of vascular permeability, promotion of neuronal apoptosis, and IL-1β production. MF enrichment indicated involvement in caspase activity, cytokine receptor interactions, and endopeptidase activity. CC analysis showed that the genes were mainly associated with inflammasome and caspase complexes, as well as tight-junction structures. KEGG analysis further demonstrated significant enrichment in immune- and barrier-related pathways, including the NOD-like receptor signaling pathway, C-type lectin receptor signaling pathway, and tight junction pathway. Collectively, these findings suggest that the intersecting genes play central roles in maintaining BBB integrity, modulating immune responses, regulating neuronal apoptosis, and mediating inflammatory cytokine production.

**Figure 5.**
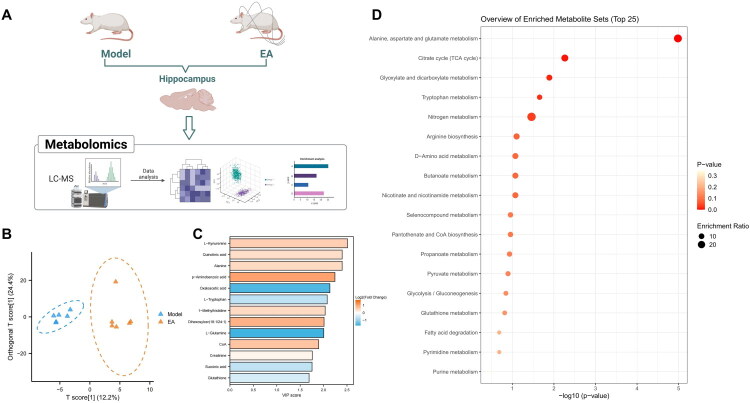
Untargeted metabolomics analysis of the metabolic mechanisms underlying the effects of EA in METH withdrawal-induced depression. (A) Schematic diagram of untargeted metabolomics based on LC-MS of hippocampal tissues from Model and EA groups; N = 6 (Created in BioRender); (B) OPLS-DA score plot showing metabolic profile separation between groups; (C) Volcano plot of DMs with VIP > 1 and *p <* 0.05; (D) Pathway enrichment analysis of DMs based on the MetaboAnalyst database.

We subsequently collected hippocampal tissue from the Model and EA groups for untargeted metabolomics analysis to explore metabolic alterations associated with the antidepressant effect of EA in METH withdrawal rats. A total of 238 metabolites were detected (Figure 5A). The OPLS-DA score plot revealed a clear separation between the groups, indicating that EA significantly impacted hippocampal metabolic profiles (Figure 5B). Based on VIP values and *p*-values, 13 DMs were identified (Figure 5C). MetaboAnalyst pathway enrichment showed that these DMs were predominantly associated with alanine, aspartate, and glutamate metabolism; the TCA cycle; glyoxylate and dicarboxylate metabolism; tryptophan and nitrogen metabolism; arginine biosynthesis; D-amino acid and butanoate metabolism; and nicotinate and nicotinamide pathways (Figure 5D). These enriched pathways are broadly involved in key physiological processes such as neurotransmitter synthesis and degradation, energy metabolism, nitrogen metabolism, inflammatory cytokine regulation, and redox homeostasis. Dysregulation of these pathways may contribute to BBB dysfunction, activation of neuroinflammation, and neurotransmitter imbalance, all of which are implicated in the development of depression following METH withdrawal.

Collectively, both transcriptomic and metabolomic analyses indicated that EA effectively restored the expression of BBB-related genes and corrected neuroinflammation-associated metabolic disturbances, thereby exerting neuroprotective effects.

### The NLRP3 Activator Reversed the Beneficial Effects of EA on Depression-Like Behavior and BBB Function in Rats following METH Withdrawal

**Figure 6.**
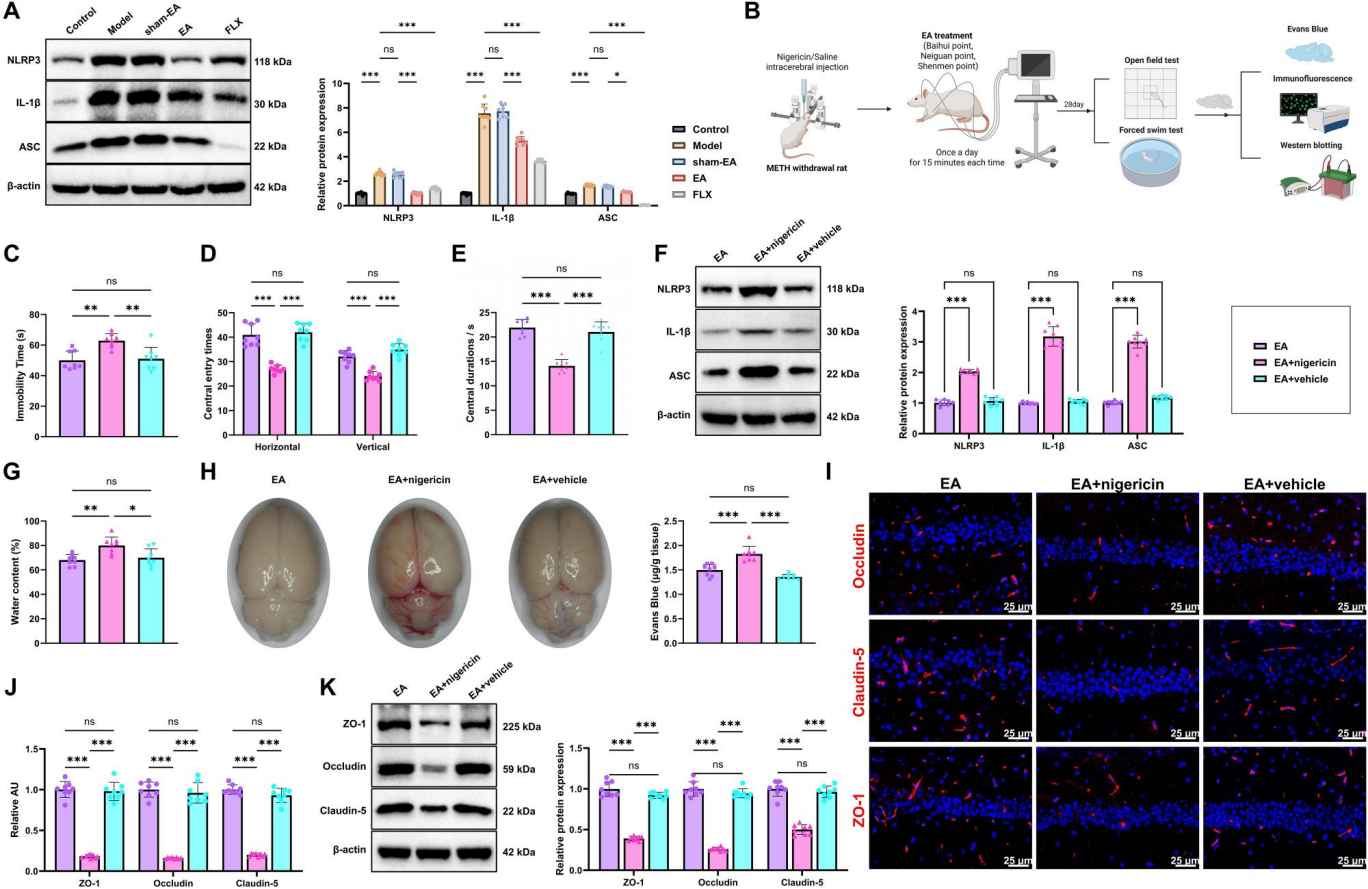
Reversal effect of the NLRP3 activator nigericin on the beneficial impact of EA on depression-like behavior and BBB function in METH withdrawal rats. (A) Western blot analysis of NLRP3, ASC, and IL-1β protein expression in hippocampal tissue; (B) Schematic diagram of the intervention protocol involving intracerebroventricular administration of the NLRP3 activator nigericin (Created in BioRender); (C) FST assessing immobility time; (D-E) OFT measuring central zone activity time (D), rearing frequency, and total locomotor score (E); (F) Western blot analysis of NLRP3, ASC, and IL-1β protein levels in the hippocampus; (G) Hippocampal water content measured by the dry-wet method; (H) Evans Blue extravasation assay evaluating BBB permeability; (I-J) Immunofluorescence staining for BBB structural proteins Occludin, Claudin-5, and ZO-1 in hippocampal tissue; scale bar: 25 μm; (K) Western blot analysis of Occludin, Claudin-5, and ZO-1 protein levels in hippocampal tissue. N = 8; *p < 0.05; **p < 0.01; ***p < 0.001 between groups.

WB analysis revealed that hippocampal NLRP3, ASC, and IL-1β levels were markedly elevated in the Model group relative to the Control group. These elevations were significantly reduced following EA or FLX treatment, while the sham-EA group exhibited no notable changes (Figure 6A). These findings corroborate the RNA-seq results and further indicate that EA exerts its antidepressant effects after METH withdrawal by suppressing NLRP3 inflammasome activation.

To determine whether the therapeutic effect of EA could be reversed by activating the NLRP3 pathway, we administered the NLRP3 activator nigericin via intracerebroventricular injection 2 hours before EA treatment (Figure 6B). In the FST, rats in the EA + nigericin group exhibited a significant increase in immobility time compared with the EA group, while the EA + vehicle group showed no significant difference (Figure 6C). In the OFT, rats in the EA + nigericin group exhibited significantly reduced central zone activity time, rearing frequency, and total locomotor score relative to the EA group, while the sham-EA group displayed no notable change (Figure 6D-E).

Following euthanasia, hippocampal tissues were collected for further analysis. WB results showed that the EA + nigericin group had significantly higher hippocampal expression of the NLRP3 inflammasome–related proteins NLRP3, ASC, and IL-1β compared with the EA group, whereas the EA + vehicle group showed no meaningful differences (Figure 6F). Consistently, hippocampal water content was significantly higher in the EA + nigericin group than in the EA group, while the EA + vehicle group showed no detectable change (Figure 6G). TEvans Blue extravasation assays further demonstrated that BBB permeability was markedly increased in the EA + nigericin group relative to the EA group, with no significant variation in the EA + vehicle group (Figure 6H). Immunofluorescence and WB analyses revealed that expression levels of the BBB tight junction proteins Occludin, Claudin-5, and ZO-1 were significantly reduced in the EA + nigericin group compared with the EA group, while the EA + vehicle group showed no significant difference (Figure 6I-K).

These findings demonstrated that the NLRP3 activator nigericin effectively reversed the beneficial effects of EA on depression-like behavior and BBB function in rats following METH withdrawal.

### The NLRP3 Activator Reversed the Inhibitory Effects of EA on Neuronal Apoptosis, Microglial Activation, and Inflammatory Cytokine Levels in Rats following METH Withdrawal

**Figure 7.**
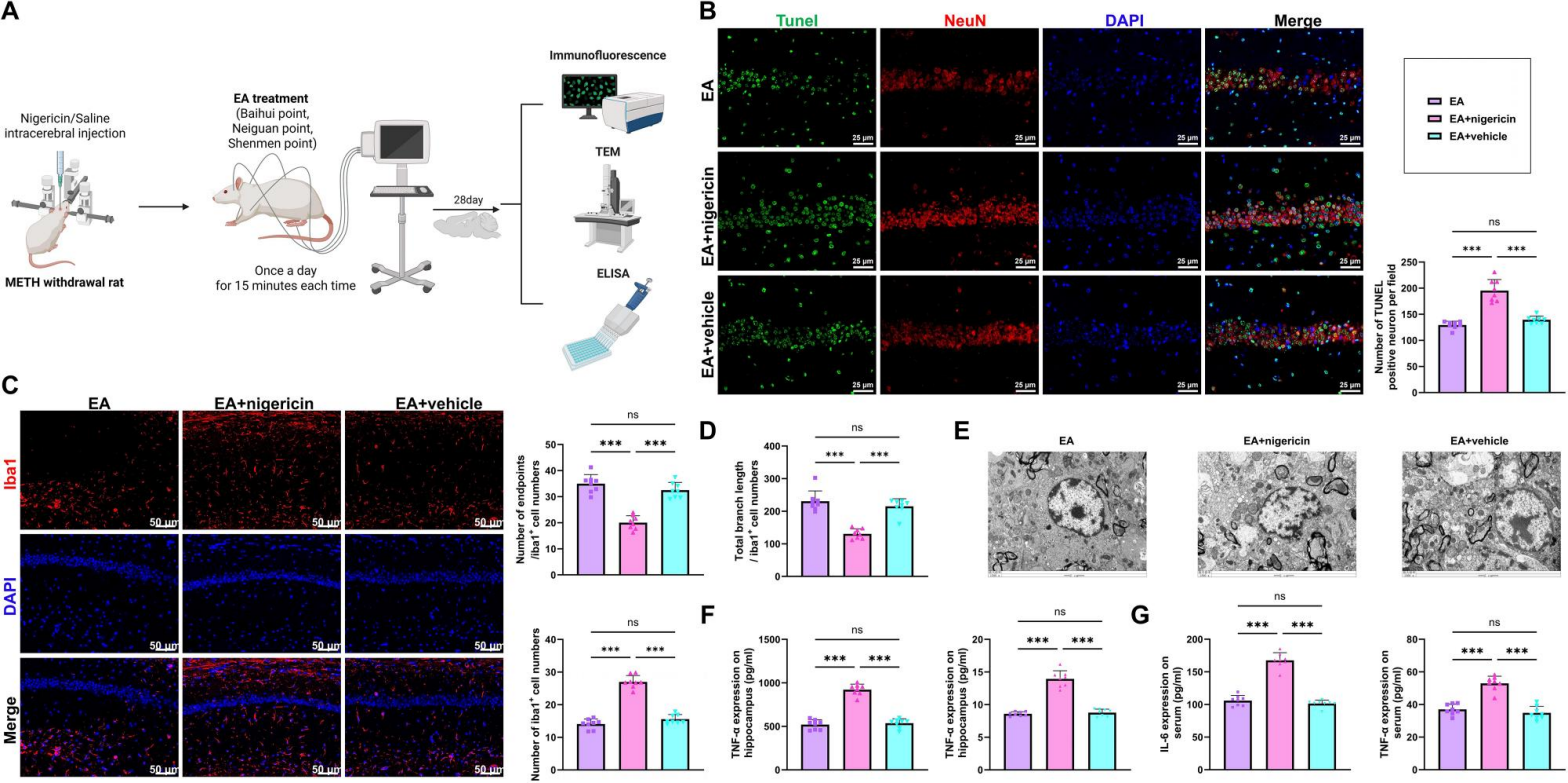
Reversal effect of the NLRP3 activator nigericin on the inhibitory action of EA on neuronal apoptosis, microglial activation, and inflammatory cytokine levels in METH withdrawal rats. (A) Schematic diagram illustrating the experimental design for assessing the reversal effect of nigericin on the neuroprotective actions of EA (Created in BioRender); (B) TUNEL staining combined with NeuN immunofluorescence to assess hippocampal neuronal apoptosis; scale bar: 25 μm; (C-D) Iba-1 immunofluorescence to quantify microglial number and analyze morphological features, including number of endpoints and total branch length; scale bar: 25 μm; (E) TEM observation of hippocampal neuronal ultrastructure; scale bar: 2 μm; (F-G) ELISA results for IL-6 and TNF-α levels in hippocampal tissue and peripheral serum. N = 8; *p < 0.05; **p < 0.01; ***p < 0.001 between groups.

We further examined whether the NLRP3 activator nigericin could reverse the inhibitory effects of EA on neuronal apoptosis, microglial activation, and inflammatory cytokine levels in METH withdrawal rats (Figure 7A). TUNEL/NeuN double staining showed that the EA + nigericin group displayed a significantly higher number of TUNEL-positive hippocampal neurons compared with the EA group, whereas the EA + vehicle group exhibited no significant difference (Figure 7B). Microglial activation was assessed using Iba-1 immunofluorescence staining. The EA + nigericin group showed a significant increase in Iba-1–positive cells in the hippocampus compared with the EA group, whereas the EA + vehicle group displayed no meaningful change. Further morphological analysis revealed that microglia in the EA + nigericin group had a significantly reduced number of endpoints and shorter total branch length compared to the EA group, indicating a more activated morphology. No comparable changes were observed in the EA + vehicle group (Figure 7C-D). TEM revealed that, compared to the EA group, the EA + nigericin group showed marked nuclear pyknosis, mitochondrial swelling, and cristae disruption in hippocampal neurons, whereas the EA + vehicle group displayed no obvious alterations (Figure 7E). ELISA results demonstrated that IL-6 and TNF-α levels in both hippocampal tissue and serum were significantly elevated in the EA + nigericin group relative to the EA group, while cytokine levels remained unchanged in the EA + vehicle group (Figure 7F-G).

Collectively, these results show that nigericin effectively abolishes the protective actions of EA by increasing neuronal apoptosis, enhancing microglial activation, aggravating mitochondrial injury, and elevating pro-inflammatory cytokines. These results highlight inhibition of the NLRP3 inflammasome as a key mechanism underlying the neuroprotective actions of EA.

## Discussion

This study systematically evaluated the therapeutic effects of EA on depression-like behaviors in rats following METH withdrawal and explored the underlying mechanisms, with a particular emphasis on the interplay between NLRP3 inflammasome inhibition and BBB restoration. The findings hold significant theoretical and clinical implications. Unlike most previous studies on METH addiction, which have primarily focused on dopaminergic imbalance, dysfunction of the reward circuitry, or psychotic symptoms (Bramness et al., 2012; Paulus and Stewart, 2020), this study is the first to introduce EA—a traditional non-pharmacological intervention—as a systematic approach to modulating negative affective states during METH withdrawal. The marked antidepressant effects observed in both the FST and OFT provide a novel non-drug-based therapeutic strategy for addiction treatment. Although prior research has reported the emotional regulatory effects of acupuncture, these findings were largely confined to models of chronic stress or brain injury (Guo et al., 2020; Pang et al., 2023). In contrast, the present study specifically targeted the unique pathological condition of METH withdrawal, lending greater specificity and innovation to the findings.

Building on the behavioral results, this study further explored the neurobiological basis by which EA ameliorates METH withdrawal depression and identified a close association with the restoration of BBB function. Evans Blue extravasation assays showed that EA markedly reduced BBB permeability, and the expression of the tight junction proteins (Occludin, Claudin-5, and ZO-1) was significantly restored, indicating effective repair of withdrawal-related BBB disruption. Previous literature has predominantly emphasized neurotransmitter dysregulation in addiction-related pathology, often overlooking the critical role of the BBB as a central-peripheral “firewall” (Hwang et al., 2025; Wang et al., 2019). This study incorporated BBB dysfunction as a key pathological component of withdrawal-induced emotional disturbances and proposed that EA exerts neuroprotective effects via barrier restoration, thus broadening the scope of neurobiological research in addiction.

Another key finding of this study is that EA exerted anti-inflammatory effects by downregulating the NLRP3 inflammasome pathway. Results from WB, ELISA, and RNA-seq consistently demonstrated that EA significantly suppressed the expression of key molecules, including NLRP3, ASC (*Pycard*), and IL-1β, indicating a potent modulatory effect on central inflammatory responses. More importantly, intracerebroventricular injection of the NLRP3 activator nigericin reversed the beneficial effects of EA, thereby establishing a causal link and confirming the dependence of its therapeutic efficacy on the NLRP3 pathway. Unlike previous studies that focused solely on changes in inflammatory cytokine levels (Coelho-Santos et al., 2015; Thomas et al., 2008), this research employed both genetic and pharmacological approaches to validate the mechanism, providing direct evidence and enhancing the scientific rigor and credibility of the findings.

Beyond inflammation regulation, this study also addressed two critical pathological processes: microglial activation and neuronal apoptosis. TUNEL assays showed a marked decrease in apoptotic neurons in the EA group, and TEM ultrastructural analysis further confirmed the neuroprotective effects of EA. In addition, Iba-1 immunofluorescence showed a marked decrease in microglial activation in the EA group. As the principal immune cells of the CNS, microglia are known to mediate inflammatory cascades and contribute to neurotoxicity when excessively activated. Compared with earlier studies that primarily observed behavioral changes or cytokine fluctuations (Cai et al., 2019), this study comprehensively assessed the neuroprotective effects of EA at the cellular, tissue, and molecular levels, thereby providing robust evidence for its multi-target regulatory mechanisms.

This study also incorporated multi-omics analyses to more comprehensively elucidate the molecular mechanisms underlying the effects of EA. RNA-seq identified a range of DEGs that were reversed by EA, including inflammasome-related factors and multiple genes encoding tight junction proteins, further supporting its regulatory role in the inflammation-barrier pathway. Concurrently, metabolomic analysis revealed 13 significantly altered metabolites enriched in pathways such as glutamate metabolism, the TCA cycle, and tryptophan metabolism. Notably, tryptophan metabolism is closely linked to 5-hydroxytryptamine synthesis, suggesting an additional potential mechanism by which EA may alleviate affective symptoms. Integrating transcriptomic and metabolomic data introduced a systems biology perspective into the mechanistic study of traditional EA, adding depth and breadth to the findings.

Compared with previous studies examining EA in models of depression, anxiety, or stress, this work introduces several important advances. First, the use of an addiction withdrawal model, a complex pathological condition with strong clinical relevance, represents a significant methodological enhancement. Second, this study is the first to propose a tripartite pathological mechanism involving inflammation, BBB disruption, and neuronal apoptosis, and to systematically demonstrate how EA mitigates upstream inflammation, preserves barrier integrity, and ultimately reduces neurotoxicity and behavioral impairments. Additionally, by incorporating both sham-EA and FLX groups, the study effectively controlled for nonspecific procedural effects, thereby strengthening the rigor and validity of the experimental design. Overall, these findings offer important theoretical evidence supporting the clinical use of acupuncture in addiction rehabilitation.

Despite these promising results, several limitations should be acknowledged. First, while the CPP model used in this study effectively evaluates drug reward effects and post-withdrawal affective states, it relies on passive drug administration (investigator-delivered injections) and Pavlovian conditioning. Consequently, it cannot fully mimic the active drug-seeking behavior, motivational decision-making deficits, and compulsive drug use characteristic of human addiction. In contrast, the self-administration model might better reflect the voluntary and self-destructive components of addiction. Nevertheless, the CPP model remains highly relevant for capturing withdrawal-associated negative emotions, such as depression, which was the primary focus of this investigation. Future studies should incorporate self-administration paradigms to further validate the efficacy of EA on voluntary drug-seeking behaviors. Second, chronic METH abuse is often associated with a hypermetabolic and catabolic state, leading to weight loss and potential muscle impairment. This raises a valid concern regarding whether the observed behavioral deficits (e.g., increased immobility in FST or reduced activity in OFT) stem from physical debilitation rather than depression-like emotional states. However, several factors in our study suggest that depression is the primary driver. First, the 10-day withdrawal period allowed for partial physical recovery, mitigating acute physiological toxicity. Crucially, our behavioral data (Figure 1D) showed that during the Post-test phase (immediately after chronic METH exposure), the Model group actually exhibited higher horizontal and vertical activity scores than controls. This confirms that METH exposure did not impair basic motor capacity or cause paralysis. The subsequent significant drop in activity observed only after withdrawal indicates a behavioral shift towards psychomotor retardation—a hallmark of depression—rather than delayed muscle wasting. Thus, the deficits likely reflect anhedonia and despair rather than pure motor failure. Nevertheless, future studies incorporating specific motor assessments, such as the rotarod or grip strength tests, would help to more rigorously dissociate motor deficits from affective symptoms. Additionally, the experiments were conducted exclusively in rats, without validation in clinical populations. Future studies should include patient samples or neuroimaging assessments to further verify EA’s therapeutic potential. Moreover, the NLRP3 inflammasome likely interacts with other immune pathways and neurotransmitter systems within complex regulatory networks; approaches such as single-cell sequencing or spatial transcriptomics may offer deeper mechanistic insights. Moreover, the generalizability of these findings should be examined using additional models, including those that account for sex differences. Finally, optimization of EA parameters, such as stimulation frequency, intensity, and treatment duration, will be essential for improving translational applicability.

## Conclusion

This study systematically revealed the neuroprotective effects of EA in METH-withdrawn depressive rats through multi-omics analysis. The results showed that EA alleviated depression-like behaviors by inhibiting the NLRP3 inflammasome pathway, restoring BBB function, and reducing neuronal apoptosis and microglial activation. For the first time, the mechanisms of EA were elucidated from inflammatory, barrier, and metabolic dimensions, providing theoretical support for acupuncture-based interventions in addiction-related mood disorders. EA may serve as a non-pharmacological strategy for inflammation-associated psychiatric diseases, including depression, anxiety, and Alzheimer’s disease. Further validation through clinical studies and precision medicine approaches is warranted to support its translational potential.

## Data Availability

The datasets during the study are available from the corresponding author on reasonable request.

## Abbreviations

BBB: Blood-Brain Barrier
BP: Biological Processes
CC: Cellular Components
cDNA: Complementary DNA
CPP: Conditioned Place Preference
DEGs: Differentially Expressed Genes
DMs: Differential Metabolites
EA: Electroacupuncture
FLX: Fluoxetine
FST: Forced Swim Test
GO: Gene Ontology
IL-6: Interleukin-6
KEGG: Kyoto Encyclopedia Of Genes And Genomes
LC-MS: Liquid Chromatography-Mass Spectrometry
METH: Methamphetamine
MF: Molecular Functions
OFT: Open Field Test
OPLS-DA: Orthogonal Partial Least Squares Discriminant Analysis
rRNA: Ribosomal RNA
RNA-seq: RNA Sequencing
SD: Standard Deviation
TCM: Traditional Chinese Medicine
TCA: Tricarboxylic Acid
TEM: Transmission Electron Microscopy
TNF-α: Tumor Necrosis Factor-α
UPLC: Ultra-Performance Liquid Chromatography
VIP: Variable Importance In Projection
χ^2^: Chi-Square
